# How 20th-century immigrants did not have to change their last names in order to become American

**DOI:** 10.1073/pnas.2614209123

**Published:** 2026-11-03

**Authors:** Joshua R. Goldstein, Guy Stecklov

**Affiliations:** ^a^ Department of Demography, University of California, Berkeley, CA 94720; ^b^ Department of Sociology, University of British Columbia, Vancouver, BC V6T 1Z4, Canada

**Keywords:** immigration, ethnicity, name changes, administrative data, language classification

## Abstract

Reports of parents’ names in Social Security Application records allow us to quantify at a population level the extent and nature of historical last name changes by immigrants. Our analysis of European, Middle Eastern, and Asian immigrants of the early to mid-20th-century finds that more than 80% kept their last names. Computational linguistic analysis shows that most of those who did change preserved their surname’s ethnic signal, leaving only about 5% of all immigrants trying to disguise their foreign origins. Of these, Jewish immigrants had the highest rate of ethnicity-concealing last name changes; Italian, Japanese, and Chinese immigrants all had the lowest. Our proposed framework helps understand the variation: name changes were rarest where ethnic community networks were densest and where passing as unmarked was least credible—as among visibly non-White immigrants. Our findings challenge the dominant assimilationist narrative that immigrants in this era had to shed their foreign identities to enter the American mainstream. “Becoming White” by adopting an English surname was not a large part of historical immigrant experience. Rather, the overwhelming majority of immigrants chose to openly preserve their ethnic origins. Our results also suggest that nominal record linkage of historical immigrant populations may be more feasible than previously assumed.

## Significance

Surname changes have long been considered integral to immigrant assimilation into the American mainstream and an obstacle for longitudinal research on immigrants. Using a newly available source—Social Security applications from 1936 to 1970—we produce population-level estimates of immigrant surname change across 11 national-origin groups from Europe, the Middle East, and Asia. Name change was rare. Those who did change mostly simplified spelling (Papadopoulos → Pappas), with only about 5% concealing their ethnic origins (Papadopoulos → Pope). These findings challenge the widespread belief that immigrants disguised their foreign origins to “become White” and open a path to linking immigrant generations across historical records.

In the past, Marilyn Monroe was born Norma Jeane Mortenson, Kirk Douglas was born Issur Danielovitch, and Howard Cosell was born as Howard Cohen. But by the time of the Carter presidency, Zbigniew Brzezinski rose to become National Security Advisor without changing his name, and today his daughter, Mika Brzezinski, hosts TV news. Has there been a fundamental change in what it means to “become American”? Or have surname changes at a population level never really been as common as popularly perceived?

In this paper, we produce population-level estimates of immigrant name changes. We take advantage of newly available Social Security application records that report parental names (1), tracking father-to-son surname transmission across generations for European, Middle Eastern, and Asian immigrants of the early to mid-20th-century. We find that more than 80% of immigrants kept their surnames intact. Of those who changed, we use methods from computational linguistics (2) to distinguish between orthographic simplifications preserving ethnic signal (about 15% of immigrants) and the rare cases (only about 5%) who adopted English-sounding names that hid their foreign origins.

Our findings of infrequent change run counter to most of the literature. Famously, H.L. Mencken referred to the “wholesale anglicization of surnames,” pointing out, “That Smith, Brown and Miller remain in first, second and third places among the surnames of New York is surely no sound evidence of Anglo-Saxon survival.” (3) Numerous journalistic, genealogical, and linguistic studies explore surname changes, with Irish (4), Greek (5), Polish (6), and Italian (7) surnames receiving particular attention, but these studies are all case studies of name changes that occurred, and ignore the population-level preservation of distinctive ethnic names. The most comprehensive study of surname changes to date uses New York City court archives of largely-Jewish name change petitions (8), but this design also ignores those who kept their names.

Our study focuses on immigrants from non-English-speaking origin countries whose integration into American society was contested in the early 20th-century: the “new” immigrants from Eastern Europe and the Mediterranean who were seen as racial “inferiors” by nativist racial theory (9, 10), the Germans and Austrians who faced hostility during the two World Wars (11), and Middle Eastern and Asian immigrants whose legal membership in American society was contested (12, 13). The assimilation of the European groups has inspired a series of histories of the form “how the ... became White” (14–16).^*^

Our study has three primary motivations. The first is to contribute to the historical debate over the extent to which European immigrants had to hide their origins in order to join the American mainstream (17, 18) and the competing narrative that they could retain their ethnic identity (19) because, in the context of the Black/White color line, they were “White on arrival” (20). This debate also has implications for the “ethnic revival” of the 1970s (21, 22). Second, we aim to understand the various competing psychological and social factors that led immigrants to keep their names even in the face of hostility, allowing for variation in the ability of immigrants to hide their foreign origins and the psychological motivations of immigrants torn between past attachments and future hopes. Finally, our estimates of surname change rates provide a benchmark for assessing whether the historical record-linkage revolution (23) can be reliably extended to immigrant populations.

## A Framework for Interpreting Surname Change.

We formalize the decision to retain or change a last name to help sort out the various competing considerations. Our approach combines Goffman’s ideas about marked and unmarked social categories (24) with Akerlof and Kranton’s economics-inspired models of identity choice (25).^†^

We imagine that an immigrant with an ethnically marked surname chooses whether to change it by weighing the material, psychological, and social costs and benefits. Formally, change occurs when

$$p(w^{\prime }-w)\;>\;B\delta +c,$$

Here *w* is the material payoff (wages, occupational access, safety) of keeping the marked, foreign-sounding name, and $w^{\prime }$ the payoff of being read instead as unmarked (e.g., after adopting an English-sounding name). Their difference $w^{\prime }-w$ is the material gain from passing. A name change captures this gain only if it succeeds, and *p* is its probability of success—of “passing” as unmarked, given appearance, accent, and other nonname signals. The left-hand side $p(w^{\prime }-w)$ is thus the expected material gain from changing: the size of the gain times the chance of realizing it. The costs of changing are on the right-hand side. *B* is the identity value of coethnic belonging, including both subjective psychological benefits and objective (e.g., network) effects. *δ* is the fraction of that belonging lost by changing the last name. Finally, *c* is the net transaction cost in time and energy of changing a surname, minus the inconvenience (e.g., having to repeat or spell it out) of retaining it.

The framework yields several predictions that structure our empirical analysis. 1) Surname change should be rare when *p* is small, even when $w^{\prime }-w$ is large, because for visible minorities, changing a name does not allow them to “pass.” 2) Holding *p* fixed, change rates should rise with the cost of remaining marked (a fall in *w* or a rise in $w^{\prime }$), which may come from group-targeted labor-market discrimination or variation in individual earnings potential. 3) Identity change rates should fall with the density and value of coethnic belonging *B*. All other things equal, surname change rates should be lower in immigrant neighborhoods and higher in small, dispersed populations. 4) Independent of their ethnic signal, name change rates should be higher for names that have high day-to-day transaction costs (e.g., long names or names one has to spell out repeatedly), lowering the net transaction cost *c* of changing a name.

## Challenges of Measurement.

The mass digitalization of census manuscripts has produced a new era of quantitative historical research, with the ability to track individuals over time using nominal record linkage (29). But censuses themselves cannot be used to study name changes because there is no reliable way to link individuals that does not use names as at least one of the matching criteria.

Historians studying name changes usually rely on official records of legal name changes (8). However, a feature of American law is that legal proceedings are not required for name changes—and it is thought that the vast majority of name changes that did occur were not recorded (8, 30).^‡^ Thus, studies of legal proceedings do not allow population-level measurement of name-change rates: both because many changers did not go to court, and because all those who kept their names are left out.

The key to measuring name change rates is thus to have a recording of every person’s original name and changed name in a single common source that does not itself rely on record linkage. Here we take advantage of a unique, large scale data source: Social Security application records (*Data* and *Materials and Methods*). These applications by workers eligible for Social Security required reporting of birth place (allowing measurement of immigrant status), own first and last name, and the first and last name of both parents.

For men, who do not typically change their last name with marriage, immigrants reporting different last names for themselves and for their father is evidence of a name change. However, concordance of names (e.g., “Smith” for father and son) does not necessarily mean there was no name change, as father and son could have immigrated together and both changed their names, or an adult son may be reporting his father’s name in an Americanized manner, regardless of whether the father also changed.

We use automated language classification methods to understand the kinds of changes we do observe and to estimate, conservatively, the changes we do not observe when father and son names changed together. For those who change their surname, we distinguish between ethnic changes—those that retain their recognizable foreign origin—and English changes that hide foreign origins. (Papadopoulos → Pappas would be an ethnic change, whereas Papadopoulos → Pope would be an English change.) Our approach uses name-frequency methods for common names and n-gram methods for rare names to distinguish English-sounding names from Ethnic-sounding names. For matched father-son names, we use similar methods to estimate the share of name changes that we do not observe directly. Details are given in *Materials and Methods*.

## Results

### Retention of Ethnically Distinctive Names.

The general pattern in the SS-5 applications is that even the most ethnic-sounding names are usually retained. When changes occur, they only rarely mask ethnic origins.

Before turning to population estimates, we begin with Table 1, which illustrates retention and change for selected common names from each ethnic origin. For Poland, we show examples of Slavic (Kowalski) and Jewish (Rosenberg) surnames. Most changes were to other ethnic names (e.g., Pappas, Papas, and Parris) rather than entirely de-ethnicized (e.g., Patterson and Pope). Even Rosenberg—a Jewish name made notorious by the Soviet-spy conviction of Ethel and Julius Rosenberg—was rarely changed, and when it was, more often to a recognizably Jewish name like Rosen or Berg than to an English name like Ross. Changes usually retain some element of father’s name: a first letter, a syllable, or a translation. Seemingly unrelated ethnic changes are sometimes because applicants carry their mother’s name (Elsner, Grundgeiger, Pikacs; marked ‡ in the table). Different naming traditions mean that some groups (e.g., Egypt) have applicants who sometimes report their father’s first name as their own last name (marked §). Across all examples, we see that the vast majority of applicants kept the name of their father: on average across the twelve origin rows, 83% of sons retained the paternal surname, 12% made an ethnic change, and only 5% adopted an English name.

**Table 1.** Examples of immigrant surname retention and change, by origin

		Son’s surname					
Origin	Father’s surname	Ethnic keepers		Ethnic changers		English changers	
Greece	PAPADOPOULOS	PAPADOPOULOS	(41)	PAPPAS	(10)	PATTERSON	(1)
				PAPAS	(2)	POPE	(1)
				PAPADO	(1)	PARRIS	(1)
			72%		23%		5%
Italy	CARUSO	CARUSO	(97)			CARR	(1)
			99%		0%		1%
Poland	KOWALSKI	KOWALSKI	(58)	KOWAL	(1)	KAY	(1)
(Slavic)			97%		2%		2%
Poland	ROSENBERG	ROSENBERG	(41)	ROSEN	(3)	OSTER	(1)
(Jewish)				ROZENBERG	(1)	ROEN	(1)
				BERG	(1)	ROSS	(1)
				ENOCH	(1)	ROSE	(1)
			80%		12%		8%
Germany	SCHMIDT	SCHMIDT	(174)	RUBARTH	(1)	SMITH	(5)
				HOFFMAN	(1)	STEVENS	(1)
				BINTZ	(1)	THONGYOO ^*^	(1)
				ELSNER ^‡^	(1)	BAXTER	(1)
				GRUNDGEIGER ^‡^	(1)		
			93%		3%		4%
Austria	SCHWARZ	SCHWARZ	(16)	SCHWARTZ	(4)	SHELDON	(1)
						STEVENS	(1)
						HAYWARD	(1)
						SCOTT	(1)
						BLAKE	(1)
			64%		16%		20%
Hungary	SZABO	SZABO	(126)	POGANY	(1)	TAYLOR	(4)
				PIKACS ^‡^	(1)		
				SARKADISZABO	(1)		
				SABO	(1)		
				KEKESSY	(1)		
			93%		4%		3%
Russia	COHEN	COHEN	(24)	KAGAN	(1)	CARLIN	(1)
				KOREN	(1)	CORWIN	(1)
				KOGAN	(1)		
				BEGUN	(1)		
			80%		13%		7%
Egypt	YOUSSEF	YOUSSEF	(7)	SAWIRIS	(1)	MARAY	(1)
				ELIAHOU ^§^	(1)		
				EFFAT	(1)		
				FARAG	(1)		
				AYOUB ^§^	(1)		
			54%		38%		8%
China	WANG	WANG	(133)	WONG	(6)	PRINCE	(1)
				YEE ^§^	(1)		
				LAI ^§^	(1)		
				KOENIG ^†^	(1)		
			93%		6%		1%
Japan	TANAKA	TANAKA	(19)				
			100%		0%		0%
Iran	EBRAHIMI	EBRAHIMI	(3)	FARDIS	(1)		
			75%		25%		0%
*Average across origins*			*83%*		*12%*		*5%*

Note: One example father surname per origin country, picked for being frequent and recognizably ethnic. The “ethnic keepers” column gives the count of sons with the same surname as the father; the remaining columns list distinct son’s surnames with counts. Percentages show the share of each type; rows sum to 100%. The bottom row is the simple mean of the twelve origin-row percentages, so each origin contributes equally regardless of sample size. Ethnic changers include shortenings, transliterations, adoption of maternal surnames (‡), and adoption of the father’s given name as a surname (§). A feature of English changers is that most preserve the first letter of the original name (Papadopoulos → Patterson, Kowalski → Kay, Schmidt → Smith, Rosenberg → Ross).

^‡^Son’s surname matches the mother’s maiden name.

^§^Son’s surname matches the father’s given name (a patronymic pattern).

^*^Thongyoo listed last name as Schmidt in 1959 but was recorded as Thongyoo in the death record.

^†^Koenig is German for “king,” a possible translation of the Chinese surname Wang, which also means king. Classified as ethnicity-retaining rather than de-ethnicizing because the son adopted a recognizably foreign (German) surname rather than an English one.

### Population-Level Rates Across 11 Origins.

**Fig. 1.**
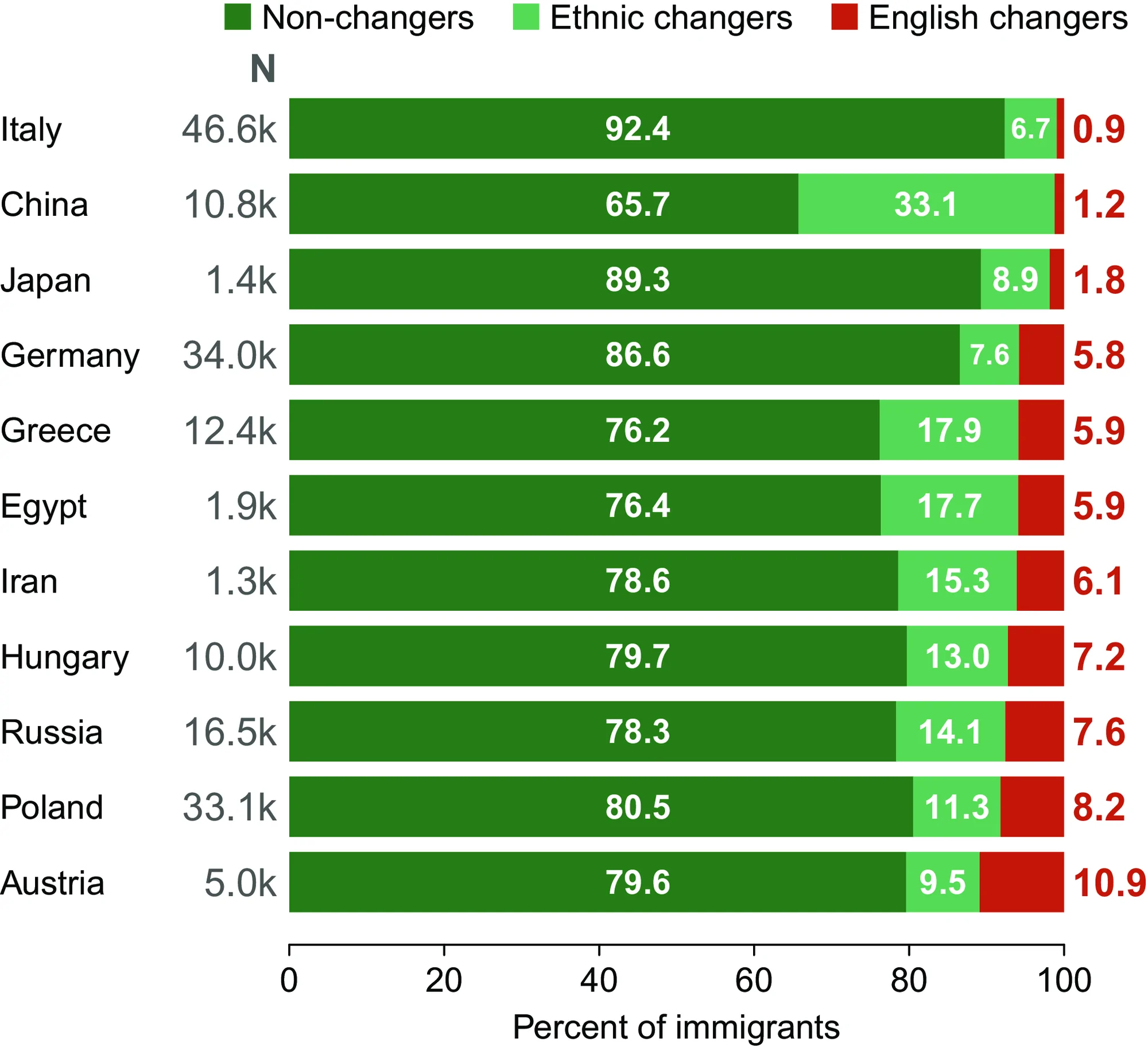
Last name changes among immigrants to the United States, 1936–1970. Countries are ordered by the rate of English changers of surnames (red bars; adjusted rate shown at *Right*). The English-changer rate is multiplied by 1.5× to account for estimated prechangers (*SI Appendix*, section S3). The large dark green bars show that the vast majority of immigrants kept their ethnic surname unchanged.

The frequencies and types of name changes for the entire population of immigrants from the 11 origin countries are shown in Fig. 1. The estimated fraction of immigrants who concealed their ethnic background is given by the red bar. This includes observed name changes to English names, where fathers and sons differ, and our estimate of same-named fathers and sons who are likely to have both changed their name (*SI Appendix*, section S3). The dark green nonchangers are the fraction of sons who report a matching ethnic last name. Light green ethnic changers are sons with ethnic names that differ from their fathers.

The lowest rates of ethnically-concealing surname changes are for Italian, Chinese, and Japanese immigrants. The highest are for Hungary, Russia, Poland, and Austria. Greece, Germany, Egypt, and Iran are all intermediate.

The pattern of name changes that retain their ethnic valence (“ethnic-changers”) is quite different from English-name changes. China and Greece have the lowest rate of identical names for father and son, because ethnic-changes are so frequent. Both languages involve originally non-Roman writing systems, which we suspect is part of the instability.

The highest rates of ethnically-concealing changes (Hungary, Russia, Poland, and Austria) are all for origins with a large fraction of Jews.

### Geography.

**Fig. 2.**
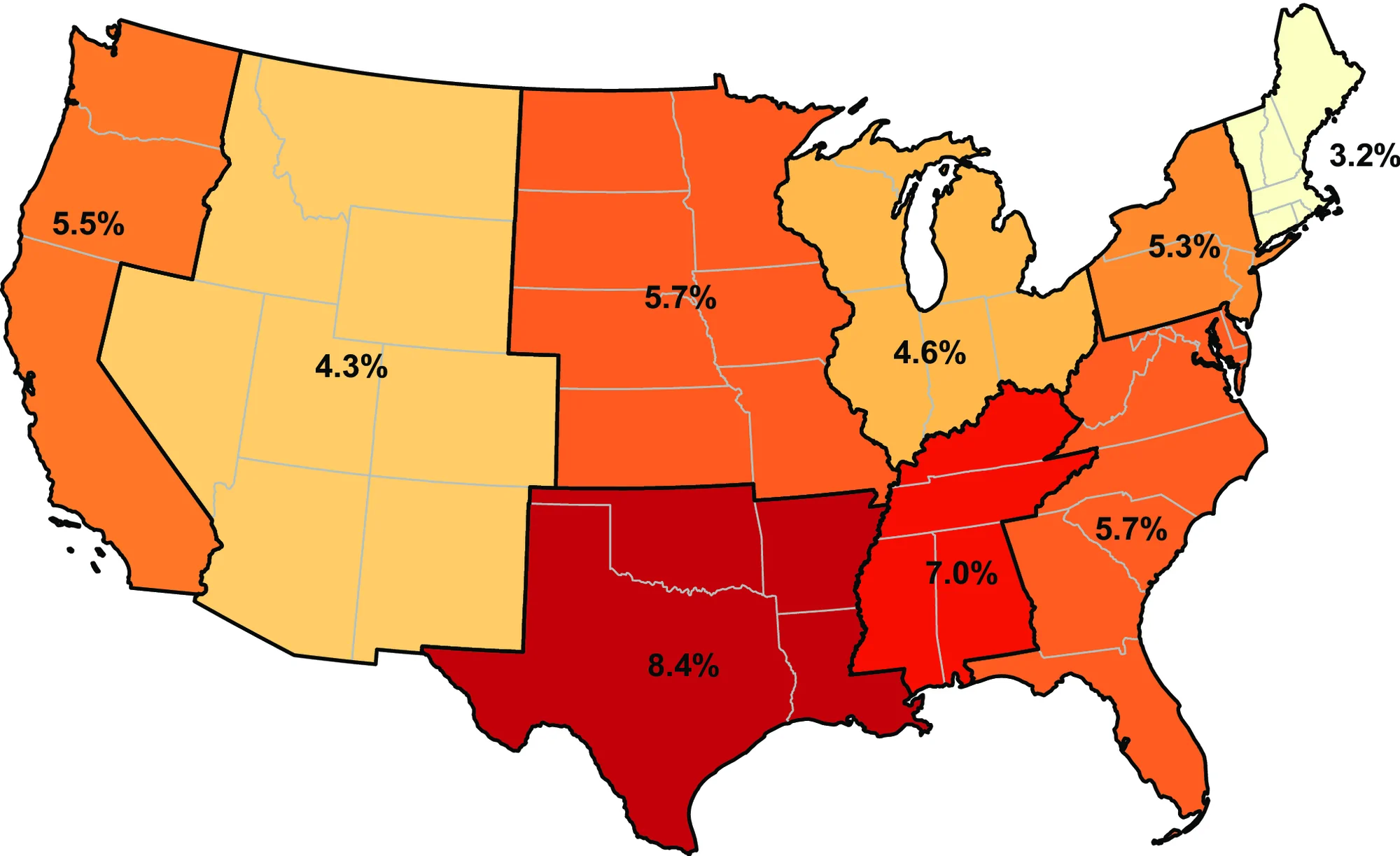
Geography of ethnicity-hiding surname changes, 1936–1970. The map shows the percentage of immigrants whose surname changed to an English-sounding name, by Census division. Rates are lowest in the Northeast gateway regions where coethnic communities were largest and highest in the South, where immigrants were fewer and the racial climate more restrictive.

Shifts to English-sounding names were rare nationwide, but rarest in the Northeast (Fig. 2), including New York City. Only 3.2% of New England immigrants concealed the foreign origin of their names. Such changes were most common in the Deep South, where the rate was more than twice as high.

### Individual and Community-Level Correlates.

**Fig. 3.**
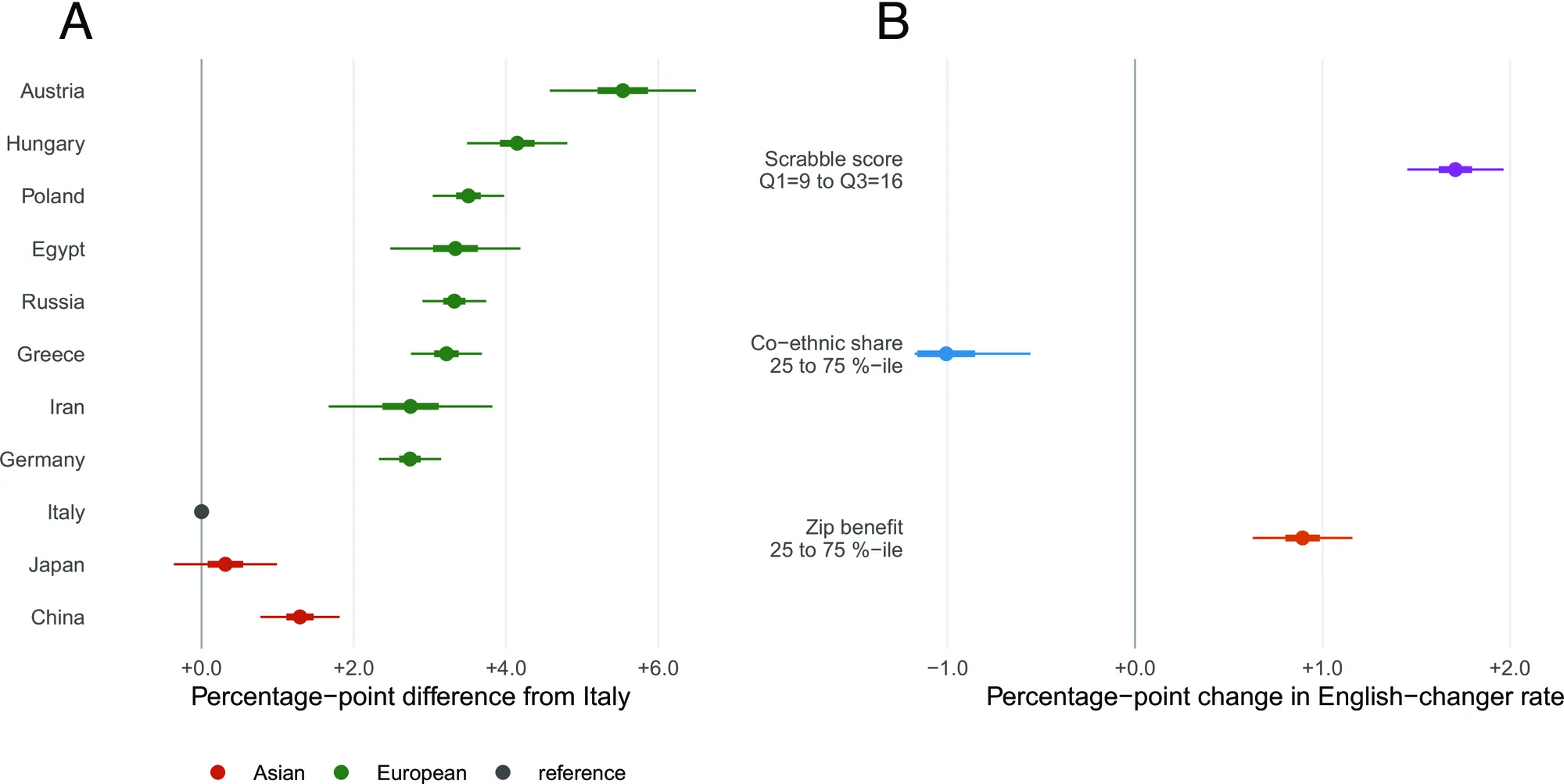
Correlates of English-surname changing among male immigrants in the BUNMD (1936–1970 SS-5 applications). (*A*) Origin effects relative to Italy, sorted within the European group; Asian origins (low *p*) shown in red. (*B*) Interquartile-range effects for the three framework-mapped covariates: Scrabble score of father’s surname (*c*), own-origin share of zip3 (*B*), and zip-level retirement benefit ($w^{\prime }-w$). Thick bars are 50% CIs, thin bars 95% CIs. Linear probability model with census-division fixed effects; SEs clustered by zip3; additional controls for time period and age of applicant. Full coefficient table and origin-specific estimates in *SI Appendix*, section S4.

The multivariate individual and community-level predictors of changing to an English-sounding name are shown in Fig. 3. The average retirement benefits of the zipcode (zipben) in which applicants live late in life proxies for earning potential. Ethnic networks are measured by the share of same-ethnic applicants living in the 3-digit zipcode area, roughly similar in size to U.S. counties. We express both zipben and coethnic share as their percentile value compared to other areas, with 0 being the bottom of the scale and 1.0 being the top. Japan and China serve as examples of visible minorities.

The average English-change rate is 3.4% for this sample.^§^ Moving from the bottom to the top of the interquartile range of zipben, our proxy for earning potential, raises the English-change rate by about 1 percentage point. In the opposite direction, living among more coethnics is associated with a lower English-name change. A higher Scrabble score for the father’s surname (31) (our proxy for day-to-day challenges of having a foreign name) raises the English-change rate by roughly 1.7 percentage points across the interquartile range. The low English-name change rates for Asian immigrants from Japan and China persist even after controlling for these other factors (see *SI Appendix*, section S4 for the full coefficient table and origin-specific heterogeneity estimates).

Finally, we briefly explore the name-changes among Jews, for whom hiding ethnic origins is thought to have been perhaps most common (8). We restrict ourselves to changes for immigrants who report their fathers as having one of 36 Distinctive Jewish Names (32)—names such as Shapiro, Goldstein, or Cohen, which are nearly exclusively held by Jews (*N* = 2,180). We find that even in this highly marked group 88.3% retain their name, 5.2% change to a different ethnically recognizable name, and only 6.5% change to an English-sounding name that hides their origins. The literature is correct in identifying Jews as among the most frequent surname changers, but misses the modal behavior—that even among immigrants with the most distinctive Jewish names, the vast majority chose to keep rather than change their surnames (see *SI Appendix*, section S5 for the DJN list and the per-origin breakdown).

## Discussion

For the vast majority of immigrants, becoming American in the 20th-century did not consist of hiding ethnic origins. Even Jews, the most frequently cited group for last name changes, ended up overwhelmingly keeping their marked names. For all groups, changes to English-sounding surnames were the rare exception, not the rule. Becoming American was a question of showing allegiance (33) while acknowledging foreign origins: adopting or being given an “American” first name and retaining an ethnic surname was the most common behavior for entering the American mainstream.

What do our results tell us about why immigrants chose to keep their names? One explanation is that Europeans, at least, were “White on arrival” (20). That is, despite the abundant rhetoric decrying immigrants as inferior, being on the White-side of the American color line was what mattered. Another is that for Asians and others who remained distinctive through appearance, accent, dress, or language, changing their surname was not enough to disguise their foreign origins. A third reason is that there were costs to changing one’s identity, both in personal, psychological terms and in terms of the benefits of embeddedness in ethnic social networks.

The formal model of identity choice provides an accounting for these factors, which our empirical results are broadly consistent with (even if not providing causal identification). The regression correlates in Fig. 3 line up with the four predictions of our framework: i) a low rate of English-changes for visible minorities who have a small chance of passing (a low *p*); ii) a lower rate of English changes for those living near coethnics (high *B*); iii) more surname changes for those whose original name had higher day-to-day costs in terms of spelling and communication; and iv) higher change rates for those with more to gain, with a larger gap between $w^{\prime }-w$. Other factors beyond what we can measure—bureaucratic costs, occupational roles, family traditions—are also likely to have played a role.

A result that is outside of our framework is the relatively high rate of English-changers in the Deep South. Extending Guglielmo’s “White on arrival” logic (20), we would expect a sharper Black/White divide to reduce the pressure on European immigrants to further differentiate themselves from other whites, predicting lower English-changer rates where the color line is most salient. The Deep South shows the opposite, suggesting that strong anti-immigrant sentiment (34) outweighed the advantage of being on the White side of the color line. Consistent with this interpretation, mid-century survey research found anti-Semitic attitudes to be strongest in the South (35).

The pattern of surname changes that did occur suggests an additional personal motivation to why such changes were so rare. Immigrant name changers usually kept some linguistic connection to their original name, such as a first letter, a translated meaning, or a shortening (Papadopoulos → Pappas; Rosenberg → Rosen). This is consistent with the idea that they were not replacing one identity with a completely different one but rather maintaining a bridge that connected their new “American” selves to their own personal family histories. We believe that this is consistent with the psychological challenges of immigrants. As Handlin recounts in his classic, The Uprooted, immigrants were no longer in the “old country” but not yet “at home.” For most, an inherent part of being an immigrant was retaining some connection to the past, even while becoming American.

Our findings also suggest a reinterpretation of the “ethnic revival” of the 1970s (21). In light of the fact that people had kept their ethnic last names, the revival was less a recovery than a rediscovery of ethnicity that was already hiding in plain sight (22).

Finally, from a methodological perspective, because immigrants mostly kept their names, nominal record linkage of historical census and administrative data is more robust than one might fear. Name changes happened, but most immigrants should be linkable from one generation to the next.

## Materials and Methods

All data is publicly available. (See *SI Appendix*, section S6 for the code repository and data-access details).

### Data.

Sons’ and fathers’ names are recorded in Social Security Applications (SS-5), available in the Berkeley Unified Numident Mortality database (BUNMD) (1). The SS-5 applications were typically made by employees to establish a Social Security number. The BUNMD contains 49.3 million records. We restrict to men whose first SS-5 application was filed between 1936 and 1970 with complete birthplace, race, residence, and parental-name fields, yielding 11.0 million applicants, of whom 596 thousand are foreign-born. Our main analysis covers the approximately 172,000 men from our 11 origins of interest (Italy, Germany, Poland, Russia, Greece, China, Hungary, Austria, Egypt, Japan, and Iran), chosen for their mix of European, Middle Eastern, and Asian origins subject to 20th-century nativist and racial discourses, and for having enough distinctive names to support origin-specific *n*-gram classifiers. *SI Appendix*, Table S1 gives the full sample selection process and per-origin counts. *SI Appendix*, section S1.1 checks that the ZIP-residence filter does not bias the English-changer rate.

The BUNMD sample relies on National Archives records consisting primarily of people dying between 1988 and 2005 and born after 1900. Most of the immigrants in the sample arrived in the United States toward the end of, or after, the great waves of migration earlier in the century. In *SI Appendix*, section S1.2 we show that the small sample of earlier immigrants (proxied with birth dates before 1900) were slightly more likely to adopt an English name. About 94% of those born before 1900 retained a recognizably ethnic surname, compared with roughly 97% for the sample as a whole.

The universe of “English” names is based on household heads in the 2% sample of the 1851 British census (29). See *SI Appendix*, section S2.1 for detail.

### Name Classification.

We use a combination of methods that allow us to classify both common and rare surnames.

For common surnames, we classify using the frequency distributions of last names from immigrants’ fathers. We define a British Surname Index of the “Englishness” of a name in the same way that the “Black Name Index” (26) and “Ethnic Name Index” (33) defined their indices for first names. For surname $S_{i}$ and ethnic origin *j* we write

$${\text{BSI}}_{\mathrm{ij}}=\frac{p(S_{i}|\text{English})}{p\left(S_{i}|\text{English}\right)+p\left(S_{i}|\text{Ethnic origin}\;j\right)},$$

where $p(S_{i}|\text{English})$ is the surname’s frequency in the 1851 British census and $p(S_{i}|\text{Ethnic origin}\;j)$ is its frequency among fathers of BUNMD applicants born in origin *j*. The index ranges from 0 for a name that occurs only in origin *j* to 1 for a name that occurs only among the English; names with ${\text{BSI}}_{\mathrm{ij}}>0.5$ are classified as English. We compute the BSI only for surnames with at least five pooled occurrences in the English plus origin-*j* reference lists; rare names are classified using the *n*-gram classifier described below. For example, the fraction of 1851 English with the surname “Pope” is 37/82 K and the fraction of Greek immigrant fathers with surname “Pope” is 1/16 K, giving ${\text{BSI}}_{\text{Pope},\text{Greece}}=0.88$.

Many surnames are rare or even unique (36). In order to classify rare names, we use computational *n*-gram-based methods. Each name is broken down into its single and multiletter parts and compared to the distribution of these parts derived from a corpus of names known to be from a given origin. The classification is based on the rank-based methods of Cavnar and Trenkle (2). For example, the name KOVEL would be broken into the n-grams up to length 4 as _K, O, V, E, L_, _KO, OV, VE, …, _KOVE, OVEL_ and the rank of these substrings compared according to each of the languages being considered for classification. The language in which the *n*-grams are more commonly found is chosen. The corpus of names we use to build *n*-gram profiles are based on the SS-5 reports of father’s names by applicant’s country of birth (with obviously English names manually removed) and the British census of 1851 (29), which has a relatively low number of immigrants.

Our goal in using *n*-grams is to have a method that can be applied to any name, regardless of its frequency, and one that resembles human classification of unknown names, with letter combinations serving as an indicator of familiarity or unfamiliarity—and possibly of a particular national or ethnic origin. Details of the reference lists (including the English-overlap cleaning step) are in *SI Appendix*, section S2.1; held-out validation of the *n*-gram branch and a misclassification correction are in *SI Appendix*, section S2.2.

### Adjusting for Unobserved Changes.

Our classification of name changes is done in two stages. Initially, we group sons’ surnames into four groups, according to a) whether father’s and applicant’s name matched and b) whether their name is classified as “English” or “non-English” using the method described above. All applicants with matching names are initially categorized in the “no change” group; all nonmatching English names are categorized as “English-changers,” and all nonmatching ethnic names are categorized as “Ethnic-changers.” In the second stage, we multiply the share of English-changers by an adjustment factor of 1.5, our upper-bound estimate of the rate of unobserved name-changes. This estimate is based on two independent estimates: i) the fraction of immigrants who arrive before age 16 (and are thus likely to have arrived with their parents and changed their name simultaneously); ii) a ratio method, which compares the ratio of unchanged English names (e.g., ROSS-ROSS) to the frequency of that same name as a target for English-changers (ethnic → ROSS). Details of the unobserved-changer adjustment are in *SI Appendix*, sections S3 and S3.3 reports robustness of the rate to the multiplier.

## Supplementary Material

Appendix 01 (PDF)

## Data Availability

Code for replicating results has been deposited in Zenodo (https://doi.org/10.5281/zenodo.21285258) (37). All study data are available: The BUNMD data underlying this analysis are available through CenSoc (https://censoc.berkeley.edu/) under their data use terms, subject to redistribution restrictions (38). The 1851 Great Britain census sample used for classifier reference lists is freely available upon registration through IPUMS International. All derived files (n-gram profiles, BSI vectors, cleaned father-son pair files, and regression-ready datasets) are generated deterministically by the Zenodo archived code. Previously published data were used for this work (1, 29, 39).

## Footnotes

^*^We leave Spanish-speaking immigrants out of the current study because different methods would be needed to account for their distinct surname practice of combining paternal and maternal last names. We also omit African-origin immigrants—who at this time were largely entering via the Caribbean and came without a single linguistic marker of their identity due to the mixed language origin of this region.

^†^Our framework also draws on Fryer and Levitt’s (26) treatment of passing probability in the context of distinctive names for Black Americans and on formal models of ethnic identity choice (27, 28).

^‡^Contrary to popular belief, immigration officials did not change immigrant names at ports of entry (8).

^§^This low average results from the heavy representation of Italians, who are a low-changing group.
