# Supplementary material for: How 20th-century immigrants did not have to change their last names in order to become American
Source: Proc Natl Acad Sci U S A. 2026 Nov 3;123(45):e2614209123. doi: 10.1073/pnas.2614209123 (PMC13751673; doi:10.1073/pnas.2614209123)
Supplement: Supplementary file 1 [file pnas.2614209123.sapp.pdf]

Supplementary Information for

From Brzezinski to Brzezinski: How 20th Century Immigrants Didn't Have to Change their Last Names in Order to Become American

Joshua R. Goldstein[*] Guy Stecklov[†]

August 21, 2026

This PDF file includes:

Supplementary text (Sections S1–S7)
(S1 BUNMD sample; S2 Name classification;
S3 Unobserved changers; S4 Regression;
S5 Distinctive Jewish Names; S6 Code and data availability;
S7 Statement on use of AI)
Figure S1
Tables S1 to S14
References

[*]Department of Demography, University of California, Berkeley. Corresponding author: josh.goldstein@berkeley.edu.

[†]Department of Sociology, University of British Columbia, Vancouver.

Contents

S1 Sample Construction in the BUNMD **3**
S1.1 Sample selectivity . . . 5
S1.2 Birth-cohort coverage and early arrivals . . . 6

S2 Name Classification **7**
S2.1 Reference Lists for the Name Classifier . . . 7
S2.1.1 English reference: 1851 Great Britain census sample . . . 7
S2.1.2 Non-English lists: BUNMD fathers' surnames . . . 7
S2.1.3 Cleaning: removing English overlap . . . 7
S2.2 Classifier Validation . . . 8
S2.2.1 How surnames are classified: BSI vs. n-gram . . . 8
S2.2.2 Held-out validation . . . 9
S2.2.3 Illustrative misclassifications . . . 10

S3 Estimating Unobserved Changers **11**
S3.1 Method 1: age at immigration . . . 11
S3.2 Method 2: the ROSS ratio . . . 12
S3.3 Sensitivity to the unobserved-changer multiplier . . . 12

S4 Regression Analysis of English-Changing **13**
S4.1 Specification . . . 13
S4.2 Full-model estimates . . . 14
S4.3 Heterogeneity by origin . . . 14

S5 Distinctive Jewish Names **15**

S6 Code and Data Availability **16**

S7 Statement on Use of AI **16**

List of Figures

S1 Example SS-5 Social Security application (Sam Devorkin, 1952); comparing the applicant's surname to the father's identifies name changes. . . . 3

List of Tables

S1 Sample selection. Each row applies one restriction to the row above; the last row is the analysis sample. Restrictions are ordered as the code applies them, and because they are all conjunctive the final sample does not depend on the order. . . . 4
S2 Foreign-born male applicants filing a first SS-5 between 1936 and 1970, by country of birth. Panel (a) is the analysis sample. Panel (b) lists origins excluded by design, with the reason. The remaining rows account for the difference, so the parts sum to the total. . . . 5

S3 English-changer rates among applicants with and without a recorded residence ZIP. The zip-missing records are classified with the classifier fitted on the zip-present data, unrefitted. A negative difference means the excluded applicants changed names less often than those retained. 6
S4 English-changer rate by birth cohort, unadjusted. Mean age at application is shown because birth cohort and application cohort are not interchangeable: later-born applicants filed younger. 6
S5 Share of each origin's n-gram training names dropped for also appearing in the England-1851 corpus, with the three most frequently dropped surnames. 8
S6 Share of changers' surnames classified by the British Surname Index (BSI) rather than the n-gram fallback, by country of birth. BSI covers a larger share of individuals than of unique surnames because common surnames recur. 9
S7 Held-out n-gram misclassification and the corrected English-changer rate. α: rate at which a truly-origin surname is labelled English; β: the reverse. $w_{n\text{-gram}}$: share of changers the n-gram decides. Hybrid rates are $w_{n\text{-gram}}$ times the held-out rates, since BSI is taken as exact. The correction is applied among changers, the population whose surnames were classified, then rescaled by each origin's changer rate to give a share of all applicants. All entries in percent. 10
S8 Held-out surnames the n-gram branch misclassifies. The left column holds truly-origin surnames labelled English (the α errors); the right holds truly-English surnames labelled as the origin (the β errors). Up to 5 of each per origin. 11
S9 ROSS ratios. Among applicants holding a curated unambiguously-English surname, the number whose surname matches their father's (a concealed-change candidate) relative to the number whose does not (an observed changer). The adjusted columns drop candidates whose mother's maiden name also classifies as English. 12
S10 English-changer rate under the range of multipliers implied by the origin-specific ROSS ratios: the lowest (Austria, 1.05×) and the highest (Germany, 1.56×) applied uniformly to every origin, alongside the 1.5× used in the main text. 13
S11 Effects on the probability of an English surname change. 14
S12 The main specification fit separately within each country of birth. Cohort and census-division fixed effects absorbed. Clustered standard errors by 3-digit ZIP in parentheses. 15
S13 The 36 Distinctive Jewish Names [1]. 15
S14 Surname-change outcomes for immigrants with a DJN father, by country of birth. . . 16

S1. Sample Construction in the BUNMD

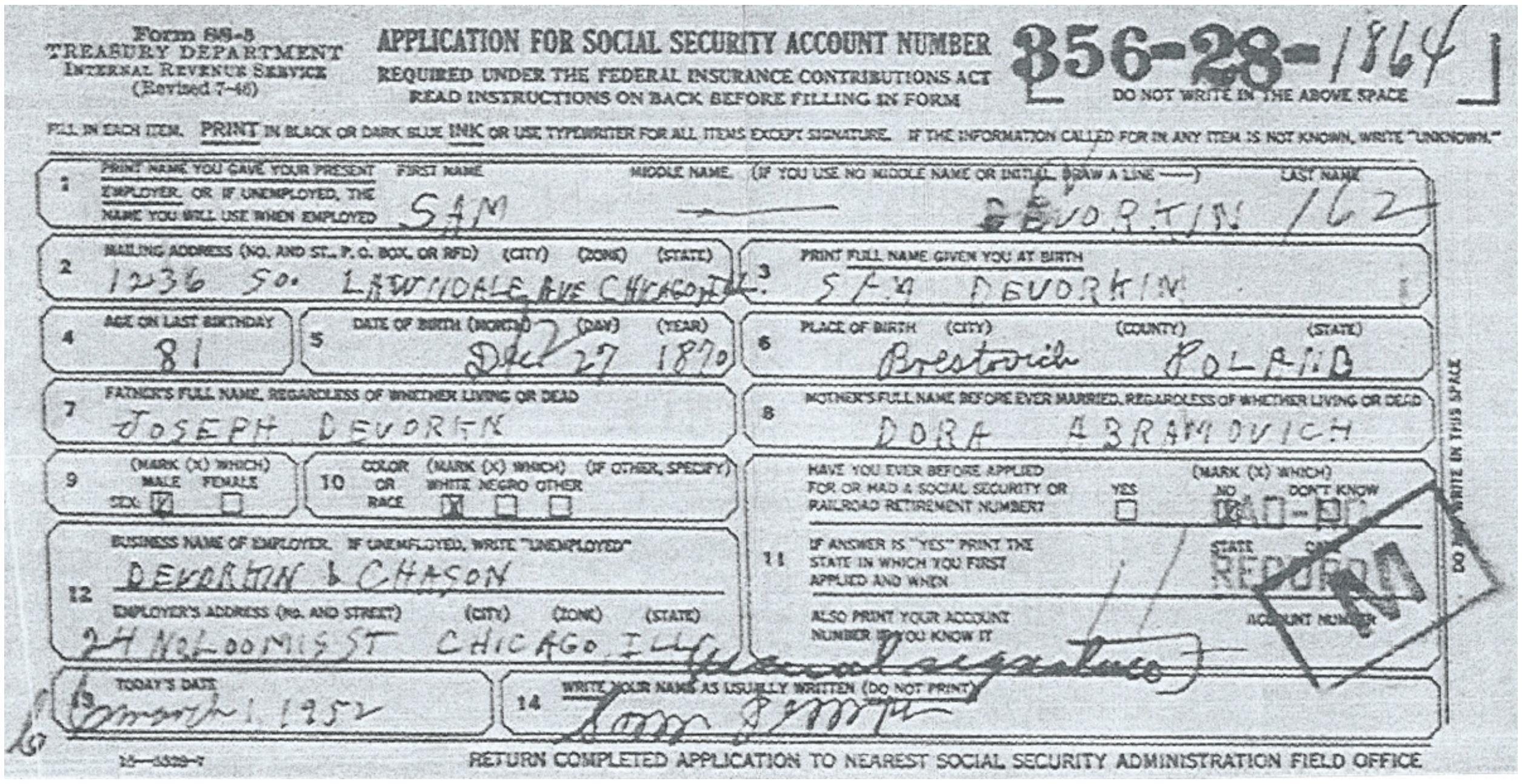
Form SS-5
TREASURY DEPARTMENT
Internal Revenue Service
(Revised 7-46)

APPLICATION FOR SOCIAL SECURITY ACCOUNT NUMBER
REQUIRED UNDER THE FEDERAL INSURANCE CONTRIBUTIONS ACT
READ INSTRUCTIONS ON BACK BEFORE FILLING IN FORM

356-28-1864
DO NOT WRITE IN THE ABOVE SPACE

FILL IN EACH ITEM. PRINT IN BLACK OR DARK BLUE INK OR USE TYPEWRITER FOR ALL ITEMS EXCEPT SIGNATURE. IF THE INFORMATION CALLED FOR IN ANY ITEM IS NOT KNOWN, WRITE "UNKNOWN."

1 PRINT NAME YOU GAVE YOUR PRESENT EMPLOYER, OR IF UNEMPLOYED, THE NAME YOU WILL USE WHEN EMPLOYED — FIRST NAME: SAM — MIDDLE NAME (IF YOU USE NO MIDDLE NAME OR INITIAL, DRAW A LINE ——) — LAST NAME: DEVORKIN 162

2 MAILING ADDRESS (NO. AND ST., P.O. BOX, OR RFD) (CITY) (ZONE) (STATE): 1236 So. LAWNDALE AVE CHICAGO ILL

3 PRINT FULL NAME GIVEN YOU AT BIRTH: SAM DEVORKIN

4 AGE ON LAST BIRTHDAY: 81

5 DATE OF BIRTH (MONTH) (DAY) (YEAR): Dec 27 1870

6 PLACE OF BIRTH (CITY) (COUNTY) (STATE): Brestovich POLAND

7 FATHER'S FULL NAME, REGARDLESS OF WHETHER LIVING OR DEAD: JOSEPH DEVORKIN

8 MOTHER'S FULL NAME BEFORE EVER MARRIED, REGARDLESS OF WHETHER LIVING OR DEAD: DORA ABRAMOVICH

9 SEX (MARK (X) WHICH) MALE [X] FEMALE [ ]

10 COLOR OR RACE (MARK (X) WHICH) WHITE [X] NEGRO [ ] OTHER [ ] (IF OTHER, SPECIFY)

HAVE YOU EVER BEFORE APPLIED FOR OR HAD A SOCIAL SECURITY OR RAILROAD RETIREMENT NUMBER? (MARK (X) WHICH) YES NO DON'T KNOW

11 IF ANSWER IS "YES" PRINT THE STATE IN WHICH YOU FIRST APPLIED AND WHEN — STATE
ALSO PRINT YOUR ACCOUNT NUMBER IF YOU KNOW IT — ACCOUNT NUMBER

12 BUSINESS NAME OF EMPLOYER. IF UNEMPLOYED, WRITE "UNEMPLOYED": DEVORKIN & CHASON
EMPLOYER'S ADDRESS (NO. AND STREET) (CITY) (ZONE) (STATE): 24 N. LOOMIS ST CHICAGO ILL

13 TODAY'S DATE: March 1, 1952

14 WRITE YOUR NAME AS USUALLY WRITTEN (DO NOT PRINT)

DO NOT WRITE IN THIS SPACE

RETURN COMPLETED APPLICATION TO NEAREST SOCIAL SECURITY ADMINISTRATION FIELD OFFICE

Figure S1: Example SS-5 Social Security application (Sam Devorkin, 1952); comparing the applicant's surname to the father's identifies name changes.

The Berkeley Unified Numident Mortality Database [2] contains 49,337,827 SS-5 administrative records. These records were transcribed by the Social Security Administration as part of their NUMIDENT database. Records of those who had died were released by the National Archives in electronic form. The BUNMD is an integrated version of all of these records.

Figure S1 shows an example of an SS-5 form for Sam Devorkin, reproduced from the Berkshire Family History Society (`https://berksfhs.org/us-social-security-number-application-forms-ss-5/`). Such forms were transcribed over decades as part of the internal Social Security Administration computerized NUMIDENT database.

We obtain our analytic sample by applying a series of restrictions, reported in full in Table S1. We restrict to men, to avoid surname changes upon marriage; to applicants whose first SS-5 was filed between 1936 and 1970, covering the period from the inception of the Social Security program through the end of our main analysis; to those with a recorded birthplace, race, US residence ZIP code, and state of application; and to those reporting their own, their father's and their mother's surname. We then restrict to the foreign-born from 11 origins and require that all three surnames survive cleaning. The number of records falls over time, because released records are for the deceased.

Finally, we exclude Black applicants from all origins and White applicants from Japan and China, as these appear often to be children of American military personnel and expatriates born abroad rather than immigrants. These restrictions are conjunctive, so the analytic sample does not depend on the order in which they are applied.

Table S1: Sample selection. Each row applies one restriction to the row above; the last row is the analysis sample. Restrictions are ordered as the code applies them, and because they are all conjunctive the final sample does not depend on the order.

Restriction	N	% of previous
BUNMD records	49,337,827	—
Men	25,769,568	52.2
Birthplace and race recorded	18,307,957	71.0
First SS-5 filed 1936–1970	15,182,059	82.9
Residence ZIP recorded	11,096,763	73.1
US ZIP prefix	10,867,628	97.9
Applied from a state or DC	10,748,933	98.9
Own, father's and mother's names recorded	10,739,736	99.9
Born in one of the 11 analysis origins	180,262	1.7
Surnames usable after cleaning	175,713	97.5
After race-based exclusions	172,939	98.4

Table S2 shows how the foreign-born applicants are distributed across countries of birth. The main analysis covers 11 non-English-speaking origins from Europe, the Middle East, and Asia. The remaining foreign-born applicants fall into categories that would require different methods: Spanish-speaking origins (Mexico, Cuba, Philippines), majority-English origins (Canada, England, Ireland), small-N European origins for which we did not build n-gram profiles (Portugal, Czechoslovakia, Netherlands), or multi-ethnic groupings (Yugoslavia).

Table S2: Foreign-born male applicants filing a first SS-5 between 1936 and 1970, by country of birth. Panel (a) is the analysis sample. Panel (b) lists origins excluded by design, with the reason. The remaining rows account for the difference, so the parts sum to the total.

Country of birth	N	Reason for exclusion
(a) Analysed		
Italy	46,626	
Germany	34,047	
Poland	33,065	
Russia/USSR	16,451	
Greece	12,370	
China	10,819	
Hungary	10,004	
Austria	4,987	
Egypt	1,902	
Japan	1,388	
Iran	1,280	
Subtotal	172,939	
(b) Excluded by design		
Mexico	65,843	Spanish surname convention (paternal + maternal)
Canada	57,614	Majority English-speaking
Cuba	43,142	Spanish surname convention
England	29,178	Reference population defining “English”
Ireland	11,030	English-speaking
Philippines	10,261	Hispanic-origin naming following Spanish colonization
Yugoslavia	9,454	Heterogeneous multi-ethnic origin
Portugal	8,557	No n-gram profile built; small analytic cell
Czechoslovakia	7,262	No n-gram profile built; small analytic cell
Netherlands	6,360	Dutch name patterns overlap German and English
Other origins	71,178	Not individually tabulated
US territory	6,817	No census division
No usable US ZIP	230,844	Residence not identifiable
**Total foreign-born**	730,479	

S1.1 Sample selectivity

Our analytical sample requires the ZIP-residence requirement, dropping 27% (4.09M of 15.18M male 1936–1970 SS-5 records), compared with <1% each for race, birthplace, socstate, or parent-name missingness. If zip-missing applicants were disproportionately name-changers, our analytic sample would understate the true rate. To check this, we select without the ZIP filter and apply the same BSI+n-gram classifier (trained on the zip-present data) to the zip-missing records. Table S3 shows the result: across every origin except Italy (flat) and two small origins (Japan, where the difference is negligible), the zip-missing group has a *lower* observed English-changer rate than the analytic sample. Dropping zip-missing applicants appears to slightly *inflate* the English-changer rate.

Table S3: English-changer rates among applicants with and without a recorded residence ZIP. The zip-missing records are classified with the classifier fitted on the zip-present data, unrefitted. A negative difference means the excluded applicants changed names less often than those retained.

	ZIP present		ZIP missing		
Origin	N	%	N	%	Difference
Austria	4,987	7.26	1,589	4.91	-2.35
Poland	33,065	5.46	7,030	4.20	-1.27
Greece	12,370	3.91	3,515	2.67	-1.24
Hungary	10,004	4.83	2,723	3.75	-1.08
Iran	1,280	4.06	596	3.19	-0.87
Germany	34,047	3.87	13,222	3.20	-0.67
Russia	16,451	5.06	3,913	4.70	-0.36
China	10,819	0.80	2,786	0.57	-0.23
Egypt	1,902	3.94	633	3.79	-0.15
Italy	46,626	0.62	11,071	0.61	-0.01
Japan	1,388	1.22	785	1.27	+0.05
Pooled	172,939	3.36	47,863	2.74	-0.62

S1.2 Birth-cohort coverage and early arrivals

Because the BUNMD includes only records of the deceased, primarily those who died between 1988 and 2005, the analytic sample is almost entirely immigrants born after 1900: fewer than 0.2% of the men in our 11 origins were born before 1900, and 98.7% were born in 1910 or later (Table S4). Earlier-born immigrants enter the sample only if they survived to advanced ages—a man born in 1890 would have been 98–115 years old during the death-coverage window—so the peak "great wave" arrivals of 1900–1914, who were typically already adults by then, are under-represented. The 19th-century-born are a small, mortality-selected group ($N = 300$).

Despite this thin coverage of the earliest arrivals, we can still characterize name-changing across the birth cohorts we do observe. The English-changer rate declines with year of birth, from 6.1% among those born in 1900 or earlier to 1.9% among those born in the 1930s (raw rates; Table S4).

Table S4: English-changer rate by birth cohort, unadjusted. Mean age at application is shown because birth cohort and application cohort are not interchangeable: later-born applicants filed younger.

Birth cohort	N	English changers (%)	Mean age at SS-5
1871-1900	363	6.06	57.3
1901-10	2,815	5.15	45.6
1911-20	72,605	4.14	31.5
1921-30	66,882	3.00	27.3
1931-40	22,646	1.92	23.4
1941-50	5,854	2.51	18.3

S2. Name Classification

We classify common names (those appearing 5 or more times) based on the relative frequencies of these names among immigrants in our Social Security applications as compared to the English reference population from 1851 Great Britain. The subsections below describe how the reference lists were built and cleaned, and report a held-out validation of the classifier.

S2.1 Reference Lists for the Name Classifier

Both the British Surname Index (BSI) and the n-gram classifier described in the Materials and Methods section of the main text require a reference list of English-sounding surnames and, for each non-English origin, a list of ethnic surnames. This section describes how those lists were built and cleaned.

S2.1.1 English reference: 1851 Great Britain census sample

For the English reference we use the surnames of household heads in the 1851 Great Britain 2% national sample [3], accessed through IPUMS International.[1] We chose our source for English names from this early census rather than looking directly at immigrants from England, because many of the immigrants from England were Jewish, Polish, and Eastern European migrants who first lived in England, and then migrated to the United States, carrying names like COHEN or KOWALSKI rather than SMITH. The 1851 sample largely predates those migrations and captures surnames of long-term English residents. The resulting list contains 81,552 surnames.

S2.1.2 Non-English lists: BUNMD fathers' surnames

For each of the 11 non-English origins, we build the reference lists from the surnames of immigrant applicants' *fathers*, restricted to applicants whose birthplace matches the origin of interest.

S2.1.3 Cleaning: removing English overlap

A fraction of surnames in the raw origin-specific lists are the "unobserved changers" of Section S3: families who reported the same English surname for both father and son. Without cleaning, surnames like SMITH, MILLER, or BROWN would be present in both the English profile and in (say) the German profile, weakening the n-gram classifier's ability to identify a German-origin SMITH as an English-changer.

We clean the non-English origin surname lists by removing names that appear in the 1851 Great Britain census. The share removed varies by origin (Table S5).

[1] We restrict to household heads to avoid duplication across family members.

Table S5: Share of each origin's n-gram training names dropped for also appearing in the England-1851 corpus, with the three most frequently dropped surnames.

Origin	Names	Dropped	%	Most frequent
Italy	46,626	386	0.8	PACE, MARTIN, BASTONE
Greece	12,370	340	2.7	GEORGE, COHEN, THOMAS
Poland	33,065	1,302	3.9	COHEN, MILLER, LEWIN
Japan	1,388	79	5.7	LEE, OHARA, ALLEN
Russia	16,451	954	5.8	COHEN, STEIN, MILLER
Egypt	1,902	129	6.8	COHEN, LEVY, ELIAS
Hungary	10,004	720	7.2	KISS, KLEIN, SIMON
Austria	4,987	526	10.5	KLEIN, STERN, FISCHER
Germany	34,047	4,346	12.8	MEYER, FISCHER, STERN
Iran	1,280	171	13.4	BENJAMIN, DOHERTY, MURPHY
China	10,819	2,503	23.1	LEE, CHAN, YOUNG

The n-gram classifier is applied to BUNMD applicants using the cleaned profiles. The original lists are used for the frequency-based approach described below.

S2.2 Classifier Validation

Our classifier assigns applicants whose surnames do not match their father's surnames to be either ethnic-changers or English-changers. The English-changer rate reported in the main text depends on the accuracy of that assignment. This section quantifies classifier accuracy and shows how inaccuracy affects our main results.

S2.2.1 How surnames are classified: BSI vs. n-gram

Table S6 reports the BSI/n-gram split by origin, restricted to mismatched changers (the population whose son-surname classification drives the English-changer rate). The BSI covers a larger share of individuals than of unique names, because common surnames are repeated many times. For Chinese changers the BSI handles 90% of individuals (though only 48% of unique names); for long-tail origins such as Greece, Russia, Japan, and Iran, the BSI handles fewer than 18% of individuals and the n-gram branch does nearly all of the work. Because the BSI operates on observed population frequencies in a large reference list, we assume that the BSI classifications are accurate, with misclassification risk coming from n-gram classification.

Table S6: Share of changers' surnames classified by the British Surname Index (BSI) rather than the n-gram fallback, by country of birth. BSI covers a larger share of individuals than of unique surnames because common surnames recur.

	Individuals		Unique surnames	
Origin	N	BSI (%)	N	BSI (%)
China	3,663	90.0	570	48.1
Germany	3,917	34.5	3,061	22.2
Italy	3,416	32.6	2,933	25.6
Hungary	1,788	29.6	1,456	20.7
Austria	836	27.6	747	23.0
Poland	5,531	25.5	4,478	15.5
Egypt	412	17.5	347	15.0
Greece	2,698	15.5	2,267	8.5
Russia	3,146	13.8	2,864	9.7
Japan	140	12.9	135	11.1
Iran	248	8.9	238	7.6

S2.2.2 Held-out validation

The classifier makes mistakes, and those mistakes are not symmetric. For most origins it is somewhat more likely to mistake a foreign surname for an English one than the reverse; and, more importantly, because the great majority of immigrants kept a foreign-sounding name, the pool of foreign names that could be mislabeled English is far larger than the pool of English names that could be mislabeled foreign. Both forces push in the same direction: the raw counts *over-state* English-changing. We measure this error directly and correct for it, and the correction lowers the estimated English-changer rate for every origin — so the rates reported in the main text are conservative.

We measure the error with a hold-out test, run separately for each origin. Take Greece. We set aside a random 10% of the distinct Greek surnames and 10% of the distinct England-1851 surnames as a test set, rebuild the two language profiles on the remaining 90%, and ask the classifier to label each held-out name as "English" or "Greek," comparing its guess to the truth. (Because BUNMD "origin" is country of birth rather than ancestry, we first drop any origin surname that also appears in the English list — for example, the son of an American family living abroad — which would otherwise count as "truly origin" and inflate the error.)

The test gives two error rates:

- α: how often a truly-origin surname is mislabeled English (a false English-change), and
- β: how often a truly-English surname is mislabeled as the origin.

For Greece, $\alpha \approx 10\%$ and $\beta \approx 4\%$ (overall accuracy $\approx 94\%$): the classifier errs both ways, but more often toward calling a Greek name English.

Two steps turn these rates into a correction. First, the n-gram is only a fallback: common surnames are classified by the exact British Surname Index (BSI) frequency lookup, and only rarer names reach the n-gram, so its errors apply to just the share it handles, $1 - w_{\text{BSI}}$ (treating the BSI branch as exact):

$$\alpha_{\text{hybrid}} = (1 - w_{\text{BSI}})\,\alpha, \qquad \beta_{\text{hybrid}} = (1 - w_{\text{BSI}})\,\beta.$$

Second, we re-estimate the observed English-changer share $\hat{p}$ with the standard [4] correction, which subtracts the false positives and adds back the false negatives:

$$p_{\text{corr}} = \frac{\hat{p} - \alpha_{\text{hybrid}}}{1 - \alpha_{\text{hybrid}} - \beta_{\text{hybrid}}}.$$

For Greece this lowers the ethnicity-hiding share among changers from about 18% observed to about 11% corrected.

Table S7 reports α, β, and w_{BSI} for every origin, together with the observed and corrected English-changer shares both among changers (p_{obs}, p_{corr}) and as a percentage of all immigrants (multiplying by the origin-specific change rate, the quantity Figure 1 reports). The correction lowers the English-changer rate for every origin. This misclassification correction is separate from, and additional to, the unobserved-changer adjustment of Section S3.

Table S7: Held-out n-gram misclassification and the corrected English-changer rate. α: rate at which a truly-origin surname is labelled English; β: the reverse. $w_{n\text{-gram}}$: share of changers the n-gram decides. Hybrid rates are $w_{n\text{-gram}}$ times the held-out rates, since BSI is taken as exact. The correction is applied among changers, the population whose surnames were classified, then rescaled by each origin's changer rate to give a share of all applicants. All entries in percent.

						Among changers		All applicants	
Origin	α	β	$w_{n\text{-gram}}$	α_h	β_h	obs.	corr.	obs.	corr.
Austria	0.185	0.100	0.724	0.1341	0.0725	43.3	37.7	7.26	6.32
Poland	0.153	0.054	0.745	0.1140	0.0402	32.7	25.1	5.46	4.21
Russia	0.175	0.084	0.862	0.1505	0.0724	26.5	14.7	5.06	2.81
Hungary	0.129	0.103	0.704	0.0910	0.0726	27.0	21.4	4.83	3.83
Iran	0.152	0.062	0.911	0.1383	0.0564	21.0	8.9	4.06	1.72
Egypt	0.196	0.088	0.825	0.1615	0.0729	18.2	2.7	3.94	0.58
Greece	0.099	0.042	0.845	0.0839	0.0356	17.9	10.8	3.91	2.37
Germany	0.178	0.116	0.655	0.1165	0.0762	33.6	27.2	3.87	3.13
Japan	0.049	0.009	0.871	0.0430	0.0081	12.1	8.3	1.22	0.83
China	0.097	0.063	0.100	0.0097	0.0063	2.4	1.4	0.80	0.48
Italy	0.046	0.054	0.674	0.0310	0.0367	8.5	5.8	0.62	0.42

S2.2.3 Illustrative misclassifications

Table S8 shows a sample of surnames that the n-gram branch misclassifies on the held-out test set. The left column contains truly-origin names labelled as English (the α errors that drive the false-positive rate for English-changing); the right column contains truly-English names labelled as the origin. The misclassified names tend to be unusual and difficult to classify even by eye.

Table S8: Held-out surnames the n-gram branch misclassifies. The left column holds truly-origin surnames labelled English (the α errors); the right holds truly-English surnames labelled as the origin (the β errors). Up to 5 of each per origin.

Origin	Origin read as English	English read as origin
Austria	GOTTLIEB, CERON, BRENNER, GROSS, JANISH	BUCHAN, FRAZER, ASSETER, FRENCH, SERGEANT
China	SEAD, VONWINCKLER, BOS, MEY, HIE	BUCHAN, SCHUCK, LONG, HUNT, TOOK
Egypt	MORY, NOTRICA, RAPHEAL, BOYDER, SAVDIE	FISHER, BUCHAN, FRAZER, ADAMS, DAVID
Germany	FULTER, WINKLER, HANOW, LETTERS, NOLL	BUCHAN, FRAZER, ASSETER, FRENCH, SERGEANT
Greece	CAN, CONSTANTINE, MAKEDON, MESHULAM, JANEDY	FRAZER, ADAMS, SASSE, CAMPION, AMES
Hungary	SIFTER, LINQUIST, GAIL, WINGLER, STUCKER	BUCHAN, FRAZER, GRAY, FRENCH, SERGEANT
Iran	PINCHOT, TSCHERNOSCHT, NATHON, MCCOMISKEY, MARCAR	FRAZER, ADAMS, DAVID, SEYMOUR, SASSE
Italy	NUBILE, REALE, SKORUPSKI, PARIDIMONR, LIVE	FRAZER, COPE, CRONIN, FRENCH, CAMPBELLVISCOUNTEMYN
Japan	KWAK, PETTENGILL, OCAMPO, FULLERT	MAGUIRE, TOMS, WAKE, NAG, TEOMUSH
Poland	LICHTENTHAL, DEREN, COXKERY, DIAMAND, ATLAS	BUCHAN, FRAZER, FRENCH, MALICH, SCHUCK
Russia	WEINER, BUNSHAFT, CANTOR, GARULAY, SAEDE	BUCHAN, FRAZER, DAVID, FRENCH, SIEKLIN

S3. Estimating Unobserved Changers

Our main-text English-changer rate is built from observed father–son surname mismatches. Some families, however, changed their surname before the son's Social Security application, so father and son report the same (already-changed) name. We call these families *unobserved changers*: they appear as non-changers in our raw data but are in fact part of the same surname-changing population we want to measure. In the main text we inflate the observed English-changer rate by a uniform factor of 1.5×. This section justifies that multiplier using two independent methods.

S3.1 Method 1: age at immigration

If a father changed the family surname while his child was still in the household, the child is likely to later report the changed name for both himself and his father. Child immigrants are therefore the group whose changes we expect to be unobserved. Let c be the share of immigrants who arrived as children. Under the two baseline assumptions that (i) every such child-immigrant change is unobserved and (ii) child- and adult-immigrant families change at the same rate, the observed changer rate equals $(1 - c)$ times the true rate, so the multiplier is $1/(1 - c)$. We define "arrived as a child" as age at immigration < 16, matching the Immigration Act of 1907's threshold for unaccompanied-minor admission at Ellis Island. In the IPUMS 1930 5% sample, 33.6% of the 14.5 million foreign-born residents with a reported year of arrival were under 16 when they arrived (range across the seven European origins: 25.7% Greece to 38.1% Russia; Japan is lower at 13.0% and Egypt higher at 48.6%), giving $1/(1 - 0.336) \approx 1.5\times$.

This multiplier has a number of assumptions going in opposite directions. If some males under 16 migrated without their fathers, or if some who migrated under 16 later changed their names independently of their fathers, then we would be over-estimating the unobserved changers. If, on the other hand, some older immigrants who changed their surnames decided to report that this changed name for their father, or if during clerical transcription, the applicant's changed name were mistakenly applied to the father, then this multiplier would be under-estimating unobserved changers.

S3.2 Method 2: the ROSS ratio

An independent estimate of unobserved changes can be obtained from name frequencies. Consider a clearly English destination surname s (e.g., Ross, Davis) that appears among immigrants from origin i. Some individuals reporting s are observed changers (N_i^{obs}: father $\neq$ son, son's name is s). Others report s for both themselves and their father (N_i^{unobs}): for surnames that virtually no origin-country family would have carried, these are almost certainly unobserved changers. The ROSS ratio is $\text{RATIO}_i = N_i^{\text{unobs}}/N_i^{\text{obs}}$; a value of 0.5 implies one unobserved changer for every two observed, i.e. a 1.5× multiplier. We compiled origin-specific lists of 9–62 clearly English destination surnames from the most common observed Americanizations. Some unobserved-changer candidates are not changers at all (e.g., Americans born abroad), so we apply a *maternal filter* that drops candidates whose mother's maiden name is also classified as English. After adjustment the ratios cluster below 0.5 (median 0.38, mean 0.30), implying that for most origins the 1.5× multiplier is if anything a slight overstatement (Table S9).

Table S9: ROSS ratios. Among applicants holding a curated unambiguously-English surname, the number whose surname matches their father's (a concealed-change candidate) relative to the number whose does not (an observed changer). The adjusted columns drop candidates whose mother's maiden name also classifies as English.

		Unadjusted		Maternal-adjusted	
Origin	N_{obs}	N_{unobs}	ratio	N_{unobs}	ratio
Germany	215	186	0.87	120	0.56
Greece	70	31	0.44	28	0.40
Poland	60	37	0.62	24	0.40
Russia	29	19	0.66	11	0.38
Hungary	76	16	0.21	13	0.17
Italy	19	4	0.21	2	0.11
Austria	62	4	0.06	3	0.05

S3.3 Sensitivity to the unobserved-changer multiplier

The 1.5× adjustment is intended to be a conservative uniform value for all ethnicities, aiming to avoid too low an estimate for English-changers. To show the sensitivity of our estimates to a range of plausible values, Table S10 applies the minimum and maximum of the origin-specific Ross-ratios to all origins. The minimum from Austria is 1.05×, and the maximum (from Germany) is 1.56×. We chose 1.5× in the main estimates because it is close to the maximum and signals the appropriate amount of precision. From this table, we can see that across the full range of plausible values, English-name changing remains rare, or rarer, than we report in the main text in Figure 1.

Table S10: English-changer rate under the range of multipliers implied by the origin-specific ROSS ratios: the lowest (Austria, 1.05×) and the highest (Germany, 1.56×) applied uniformly to every origin, alongside the 1.5× used in the main text.

Origin	N	Low (1.05×)	Main (1.5×)	High (1.56×)
Austria	4,987	7.6	10.9	11.3
Poland	33,065	5.7	8.2	8.5
Russia	16,451	5.3	7.6	7.9
Hungary	10,004	5.1	7.2	7.5
Iran	1,280	4.3	6.1	6.3
Egypt	1,902	4.1	5.9	6.1
Greece	12,370	4.1	5.9	6.1
Germany	34,047	4.1	5.8	6.0
Japan	1,388	1.3	1.8	1.9
China	10,819	0.8	1.2	1.3
Italy	46,626	0.6	0.9	1.0

S4. Regression Analysis of English-Changing

The main text reports a dot-and-whisker figure (Figure 3 of the main text) summarizing correlates of English-surname changing among first-generation male immigrants in the BUNMD. This section gives (i) the full coefficient table underlying that figure, and (ii) the same specification re-fit separately within each of the 11 origins, as a heterogeneity check.

S4.1 Specification

The outcome is an indicator $y_{iz} = 1$ if immigrant i in zip-3 area z is an English-changer: father–son surname mismatch with the son's surname classified as English-sounding by the BSI and n-gram procedure of Section S2.1. Father-son matches that are classified as English are not considered English-changers in this analysis.

We estimate a linear probability model,

$$y_{iz} \;=\; \alpha_{d(z)} + \beta_1\,\text{Origin}_i + \beta_2\,\text{Cohort}_i + \beta_3\,\text{Age}_i + \gamma\,\mathbf{x}_{iz} + \varepsilon_{iz},$$

with census-division fixed effects $\alpha_{d(z)}$, origin and cohort (5-year SS-5 application bin) dummies, age at first application in decades, and three framework-mapped covariates in $\mathbf{x}_{iz}$:

- *Scrabble score* of the father's surname (proxy for transaction cost c of carrying a phonetically foreign name, following [5]);
- *Own-origin zip-3 share* (within-origin percentile rank of the co-ethnic share in the applicant's late-life zip-3; proxy for the identity-belonging parameter $B\delta$);
- *Zip benefit* (2005 OASDI retirement benefit by zip-5, pooled percentile rank; proxy for the wage-passing return $w' - w$).

Standard errors are clustered at the zip-3 level. The reference origin is Italy (the largest and lowest-changing origin) and the reference cohort is 1936–40.

S4.2 Full-model estimates

Table S11 reports the full coefficient vector. Higher zip-benefit and higher Scrabble score predict more English-changing, while a denser co-ethnic enclave predicts less. Japan and China show English-change probabilities flat or near zero relative to Italy, consistent with visible-minority status suppressing the return to passing.

Table S11: Effects on the probability of an English surname change.

	English changer	
Italy (ref.)	—	
Austria	0.055***	(0.005)
China	0.013***	(0.003)
Egypt	0.033***	(0.004)
Germany	0.027***	(0.002)
Greece	0.032***	(0.002)
Hungary	0.041***	(0.003)
Iran	0.027***	(0.005)
Japan	0.003	(0.003)
Poland	0.035***	(0.002)
Russia	0.033***	(0.002)
Application 1936–40 (ref.)	—	
Application 1941–45	−0.000	(0.002)
Application 1946–50	−0.011***	(0.002)
Application 1951–55	−0.023***	(0.002)
Application 1956–60	−0.028***	(0.003)
Application 1961–65	−0.030***	(0.003)
Application 1966–70	−0.032***	(0.003)
Age at application (decades)	0.004***	(0.001)
Father's name Scrabble score	0.002***	(0.000)
Co-ethnic share (%-ile)	−0.020***	(0.005)
Zip benefit (%-ile)	0.018***	(0.003)
Census-division FE	✓	
Mean of outcome	0.034	
R^2	0.025	
Observations	172,213	

Notes: Reference origin Italy; reference cohort 1936–40. Zip benefit is the pooled percentile rank of the 2005 OASDI retirement benefit in the applicant's 5-digit ZIP. Co-ethnic share is the within-origin percentile rank of own-origin concentration in the applicant's 3-digit ZIP. Standard errors clustered by 3-digit ZIP. $^{*}p < 0.05$, $^{**}p < 0.01$, $^{***}p < 0.001$.

S4.3 Heterogeneity by origin

As a heterogeneity check, we re-estimate the three framework-mapped covariates separately within each origin, absorbing cohort and census division as fixed effects (Table S12). Signs are broadly consistent across European origins: positive age and zip-benefit effects, negative own-share effect. For Japan and China the zip-benefit point estimate is near zero, consistent with low passing probability dampening the return to English-changing.

Table S12: The main specification fit separately within each country of birth. Cohort and census-division fixed effects absorbed. Clustered standard errors by 3-digit ZIP in parentheses.

Origin	N	Mean y	Age (decades)	Co-ethnic %-ile	Zip benefit %-ile
Italy	46,412	0.006	0.001	-0.022***	0.001
			(0.000)	(0.005)	(0.002)
Germany	33,908	0.039	0.001	0.002	0.013**
			(0.001)	(0.007)	(0.005)
Poland	32,949	0.055	0.008***	-0.042***	0.037***
			(0.002)	(0.011)	(0.006)
Russia	16,353	0.051	0.006*	-0.033**	0.022**
			(0.002)	(0.011)	(0.007)
Greece	12,319	0.039	0.001	-0.036***	0.011
			(0.002)	(0.011)	(0.006)
China	10,796	0.008	0.002*	-0.009	0.002
			(0.001)	(0.007)	(0.003)
Hungary	9,962	0.048	0.007*	-0.004	0.012
			(0.003)	(0.013)	(0.009)
Austria	4,961	0.073	0.013***	0.003	0.015
			(0.004)	(0.020)	(0.015)
Egypt	1,895	0.039	0.007	-0.023	-0.008
			(0.004)	(0.019)	(0.015)
Japan	1,386	0.012	0.004	-0.037	-0.015
			(0.003)	(0.021)	(0.011)
Iran	1,272	0.041	0.006	-0.023	-0.004
			(0.005)	(0.022)	(0.018)

Notes: $^{*}p < 0.05$, $^{**}p < 0.01$, $^{***}p < 0.001$. Origin dummies are absent by construction – each column is one origin.

S5. Distinctive Jewish Names

The main text reports name-change outcomes for immigrants whose fathers held one of 36 Distinctive Jewish Names (DJN). We use the list of [1], drawing on Sheskin (1998) and Kohs (1942): these names are empirically concentrated among American Jews with a Jewish-identification rate above 90%.

Table S13 lists the 36 names. An immigrant enters the DJN subsample if his *father*'s surname matches one of these names; we classify the son's reported surname relative to the father's using the same BSI+n-gram procedure as the main analysis.

Table S13: The 36 Distinctive Jewish Names [1].

BERMAN	GOLDSTEIN	KATZ	ROSENTHAL
CAPLAN	GOODMAN	KOHN	RUBIN
COHEN	GREENBERG	LEVIN	SCHWARTZ
EPSTEIN	GROSS	LEVINE	SHAPIRO
FELDMAN	GROSSMAN	LEVINSON	SIEGEL
FREEDMAN	JACOBS	LEVY	SILVERMAN
FRIEDMAN	JAFFE	LIEBERMAN	STERN
GOLDBERG	KAHN	ROSEN	WEINSTEIN
GOLDMAN	KAPLAN	ROSENBERG	WEISS

The main-text passage reports, for the $N = 2{,}180$ DJN-father applicants, that 88.3% retained

the father's surname, 5.2% changed to another ethnic (non-English) surname, and 6.5% changed to an English-sounding surname. Table S14 gives the breakdown by BUNMD-recorded country of birth. Over three quarters of DJN-father applicants were born in Poland, Germany, or Russia—the origin mix expected for a pre-1924 European Jewish diaspora.

Table S14: Surname-change outcomes for immigrants with a DJN father, by country of birth.

Country of birth	N	% retain	% ethnic change	% English change
Poland	800	91.5	3.8	4.8
Germany	544	87.9	4.6	7.5
Russia	384	88.0	6.0	6.0
Hungary	245	84.1	9.8	6.1
Austria	126	79.4	5.6	15.1
Egypt	38	92.1	5.3	2.6
Greece	31	87.1	0.0	12.9
Pooled total	2,180	88.3	5.2	6.5

Note: "Retain" means father and son report the same surname. Origins with fewer than 20 DJN fathers (Italy, Iran, Japan) are omitted from the rows but included in the pooled total.

The DJN list is deliberately restrictive and excludes surnames common in the broader Eastern European population (e.g., MILLER, FISCHER, KAUFMAN), so the subsample under-counts Jewish immigrants and biases our Jewish-specific estimates conservatively.

S6. Code and Data Availability

The analysis code for this paper—including the name classifier, regression pipeline, and the scripts that generate every figure and table in the main text and the Supplementary Information—is available at `https://doi.org/10.5281/zenodo.21285258`.

Two primary data sources underlie the analysis. The *Berkeley Unified Numident Mortality Database* (BUNMD) [2] is distributed by CenSoc at `https://censoc.berkeley.edu/` and is available to download free of charge, without application or registration. The BUNMD contains individual-level Social Security Numident records, including SS-5 application fields (applicant's surname, father's surname, mother's maiden name, birthplace, and race) for deceased persons. The *Integrated Public Use Microdata Series (IPUMS)* 1930 U.S. census 5% sample and the 1851 Great Britain 2% national sample [3] are distributed by IPUMS USA (`https://usa.ipums.org`) and IPUMS International (`https://international.ipums.org`) respectively.

All derived data files used by our scripts—n-gram profiles, BSI vectors, cleaned father–son pair files, and the regression-ready datasets—are generated deterministically by the code in the repository and therefore do not need to be redistributed separately. Running the full pipeline on a current BUNMD extract and an IPUMS download reproduces the figures and tables in this paper.

S7. Statement on Use of AI

We used Claude Code (Anthropic) for assistance with coding and organizing data files through GitHub. We also used Claude Opus for literature search, and both Claude Code and Claude Opus to review our writing and suggest improvements. In addition, substantial portions of the text of this Supplementary Information were drafted with Claude and then edited by us; the main text was written by us and reviewed with Claude's assistance.

The study design, the analytic choices, and the interpretations are our own. Every number reported in the main text and in this Supplementary Information is produced by the replication package deposited at Zenodo (Section S6) and can be regenerated from it. We have checked these results and are solely responsible for the content.

References

[1] BR Chiswick, The rise and fall of the American Jewish PhD. *Contemporary Jewry* **29**, 67–84 (2009).

[2] CF Breen, JR Goldstein, Berkeley Unified Numident Mortality Database: Public administrative records for individual-level mortality research. *Demographic Research* **47**, 111–142 (2022).

[3] K Schürer, M Woollard, National sample from the 1851 census of Great Britain ([computer file]) (2008) Distributed in the U.S. through IPUMS International (https://international.ipums.org).

[4] WJ Rogan, B Gladen, Estimating prevalence from the results of a screening test. *American Journal of Epidemiology* **107**, 71–76 (1978).

[5] C Biavaschi, C Giulietti, Z Siddique, The economic payoff of name Americanization. *Journal of Labor Economics* **35**, 1–39 (2017).
